# GeMI: interactive interface for transformer-based Genomic Metadata Integration

**DOI:** 10.1093/database/baac036

**Published:** 2022-06-03

**Authors:** Giuseppe Serna Garcia, Michele Leone, Anna Bernasconi, Mark J Carman

**Affiliations:** Department of Electronics, Information, and Bioengineering, Politecnico di Milano, Via Ponzio 34/5, Milano 20133, Italy; Department of Electronics, Information, and Bioengineering, Politecnico di Milano, Via Ponzio 34/5, Milano 20133, Italy; Department of Electronics, Information, and Bioengineering, Politecnico di Milano, Via Ponzio 34/5, Milano 20133, Italy; Department of Electronics, Information, and Bioengineering, Politecnico di Milano, Via Ponzio 34/5, Milano 20133, Italy

## Abstract

The Gene Expression Omnibus (GEO) is a public archive containing >4 million digital samples from functional genomics experiments collected over almost two decades. The accompanying metadata describing the experiments suffer from redundancy, inconsistency and incompleteness due to the prevalence of free text and the lack of well-defined data formats and their validation. To remedy this situation, we created Genomic Metadata Integration (GeMI; http://gmql.eu/gemi/), a web application that learns to automatically extract structured metadata (in the form of key-value pairs) from the plain text descriptions of GEO experiments. The extracted information can then be indexed for structured search and used for various downstream data mining activities. GeMI works in continuous interaction with its users. The natural language processing transformer-based model at the core of our system is a fine-tuned version of the Generative Pre-trained Transformer 2 (GPT2) model that is able to learn continuously from the feedback of the users thanks to an active learning framework designed for the purpose. As a part of such a framework, a machine learning interpretation mechanism (that exploits saliency maps) allows the users to understand easily and quickly whether the predictions of the model are correct and improves the overall usability. GeMI’s ability to extract attributes not explicitly mentioned (such as sex, tissue type, cell type, ethnicity and disease) allows researchers to perform specific queries and classification of experiments, which was previously possible only after spending time and resources with tedious manual annotation. The usefulness of GeMI is demonstrated on practical research use cases.

Database URL

http://gmql.eu/gemi/

## Introduction

**Figure 1.**
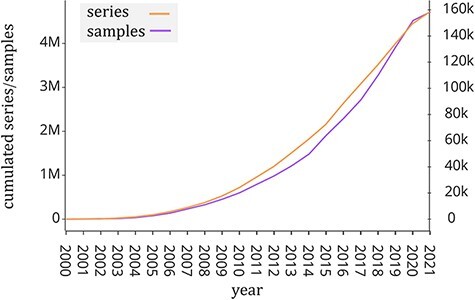
Number of samples (GSM, left *y*-axis) and experiments (GSE, right *y*-axis) made available by the GEO portal; raw data were extracted from https://www.ncbi.nlm.nih.gov/geo/browse/?view=samples and https://www.ncbi.nlm.nih.gov/geo/browse/?view=series).

Public repositories of genomic datasets such as Gene Expression Omnibus (GEO, (1)), Sequence Read Archive (SRA, (2)) and ArrayExpress (3) have become a fundamental source of knowledge that helps the scientific community to accelerate biological investigations. The analysis of its rich data corpus (including gene expression, mutation profiles and chromatin configuration) is useful to provide new insights into understanding disease and protein evolution (4). In particular, GEO is one of the largest public repositories of genomic data with >4 million experimental samples that are growing at an exponential rate in recent years (see Figure 1). This happened also thanks to next-generation sequencing technologies (5), which have greatly reduced the cost of genome sequencing. Each experimental sample contained in GEO is composed of two parts: the region data and its associated metadata. In order to classify, compare and find relevant information at scale from such a large amount of genomic data, it is essential to have a well-structured metadata content that uniquely specifies attributes such as tissue type, cell type, sex, age, disease and species.

Unfortunately, GEO metadata lack structure because they are provided in the form of a textual description of the experiment. Such text cannot be easily processed, because each information piece contained in the description may be missing, misspelled or expressed using synonyms. This issue prevents researchers from completely exploiting the knowledge contained in GEO, as the meta-analysis and the integration of multiple genomic datasets are infeasible due to the lack of machine-readable standardized metadata. For this reason, annotating genomic datasets at scale is a challenging problem for bioinformaticians (6). Three approaches are usually employed to address this problem: manual curation, metadata inference directly from gene expression profiles (or other genomic signals) and automated natural language processing (NLP). In this work, we investigated the latter, focusing on applying the last developments of deep learning transformer-based NLP. In particular, we combined Generative Pre-trained Transformer 2 (GPT2) models for attribute extraction from metadata (Cannizzaro *et al.* (7)), with the implementation of an Active Learning (AL) Framework (Cohn *et al.* (8)) and gradient-based deep learning interpretation technique (Atanasova *et al.* (9)). Recent breakthroughs in NLP with pretrained generative models as GPT2 (Radford *et al.* (10)) allowed to build multitask learners using fewer data than classic supervised Machine Learning (ML) techniques. In our work, we used GPT2 to integrate the sets of attributes provided by the datasets generated from two important genomic data sources, namely Cistrome (11) and the Encyclopedia of DNA Elements (ENCODE, (12)). However, the huge number and diversity of unlabeled samples in GEO still make it necessary to manually annotate new samples, as such data can be used to make a model able to learn dynamically over time and improve its accuracy.

AL is a meta-algorithm for ML that aims to minimize the number of samples needed to improve an ML model over time. By using an AL framework, users can verify and modify (as needed) a few specific predicted samples achieving the effect of manually annotating a multitude of samples. We designed an AL framework that learns from user feedback in real time, further reducing the number of samples to be annotated. In the last years, modern deep learning models such as Transformers (13), naturally considered as black-box techniques (i.e. not explainable), have shown an exponential growth in model parameters and computational times. This contributes highlighting the problem of user trust in the results, as—although deep learning models are able to predict correct values—these may be generated using incorrect or unethical patterns. The field of ML that addresses this issue is eXplainable AI (XAI) (14); XAI aims to make the results of complex black-box models understandable by human experts. In this work, we implemented a gradient-based technique (Kindermans *et al.* (15)) to make our model predictions interpretable by the users; we then integrated the interpretation mechanism into the AL framework; this not only improves the user experience, but also makes it easier and faster for the users to search for errors in the predictions.

The result of our work is the Genomic Metadata Integration (GeMI) web tool, which is able to extract a table composed of 15 different attributes from each selected experiment metadata of GEO. The set of attributes has been selected according to the Biological and Technological views of the Genomic Conceptual Model (Bernasconi *et al.* (16)), which recognizes a set of concepts that are supported by most genomic data sources in order to provide a common language for genomic dataset integration pipelines. The work is in line with the principles of the GeCo project (17) and sets the bases for inclusion of a relevant fraction of GEO within the META-BASE repository (18) and its search tools (19). Compared to a state-of-the-art tool for metadata annotation (Ontology Annotations and Semantic Similarity Software, OnASSiS (20)), GeMI demonstrates a considerable improvement, especially when put in the context of downstream analysis pipelines (e.g. CombSAFE (21)).

## Related work

In the last years, many works have proposed solutions for extracting structured tables from GEO data and metadata (Wang *et al.* (6)). The most straightforward approach is to manually annotate each experiment. This approach later evolved into crowd curation (Hadley *et al.* (22)), but since the amount of data is growing too fast, this approach still requires too many resources. Regarding automated techniques, we consider the work of Cannizzaro *et al.* (7), where deep learning transformer-based techniques for NLP are used to infer attributes from experiment metadata, formulating the problem as a translation task. This approach has many advantages over other classical NLP approaches such as regular expressions and classification techniques (Giles *et al.* (23), Chen *et al.* (24)); indeed, the GPT2 model does not require that the values of the attributes are known a priori (as opposed to the classification task)—it is able to handle synonyms/abbreviations and can also infer attributes from patterns (e.g. prostate implies the male sex of the donor). However, the continued growth of GEO requires a model that dynamically and efficiently learns to handle new types of experiment samples over time. This means that there is the need to collect annotations of new samples and retrain the model over time to keep it usable with new data. For this reason, the major difference of our work with respect to the available literature is the design and implementation of an AL framework that allows combining manual curation with automatic attribute extraction. In this hybrid approach, the data required to the deep learning transformer-based model are provided in an efficient way by the users during its usage. This method strategically selects a subset of the data that needs labeling to maximally improve the model performance with minimal labeling requirement. Although our model is based on a previously proposed model (7), our work differs from it because we defined the attribute extraction problem as a multitask problem where each task corresponds to a different attribute. This new formulation brings a number of advantages, the most important being that the new deep learning transformer-based model can learn to infer the union of the attributes of a multitude of heterogeneous datasets (each dataset can contain different experiment samples and different annotated attributes) and in this way it can integrate data from different data sources. Moreover, our work differs from manual curation tools (Hadley *et al.* (22)) since we implemented an interpretation mechanism for our model. This mechanism helps the user to spend less energy and time annotating samples. Finally, as other works in literature, we developed a web graphic interface that allows users to use our system to extract structured tables from GEO and annotate them without the need for programming knowledge.

Previously proposed systems (7, 25) provide scarce if not no interpretability at all. Instead, with GeMI we have made a first step toward achieving interpretability, which is paired with a functioning and effective system. On the contrary, simpler techniques (6, 23, 24) (e.g. regex-based) can easily provide interpretability by construction but suffer instead severe limitations in terms of performance.

### Use of transformers NLP-based models in bioinformatics

Several NLP techniques have been used and adapted to bioinformatics-relevant problems (26); in particular, transformer-based NLP techniques have focused on the study of protein structures (27, 28), with several approaches trying to predict other relevant genomic elements such as DNA (29), DNA enhancers (30), DNA N6-methyladenine sites (31), microRNA sequences (32) and peptides (33, 34). Few studies have addressed the problem of making the results explainable (35). In comparison, less approaches have employed transformed-based techniques for biomedical text extraction tasks (25), mainly focusing on entity relations (36, 37). To the best of our knowledge, transformed-based approaches applied to biomedical tasks have not yet been combined with explainability approaches, as proposed in this article.

## Materials and methods

### Datasets

In order to investigate the effectiveness of the aforementioned techniques in the GeMI tool, we created a new dataset that joins two heterogeneous datasets, i.e. from Cistrome (11) and ENCODE (12). The Cistrome Data Browser (11) provides a collection of 44 843 experiments data and metadata. Each sample’s metadata have been manually curated and annotated in order to provide the following attributes: Cell Line, Cell Type, Tissue Type and Factor Name. The original dataset was downloaded from http://cistrome.org/db/#/bdown. ENCODE (12) contains a collection of 20 734 experiments about DNA sequences. It provides for each experiment a set of 15 attributes. The dataset was extracted from https://www.encodeproject.org/help/batch-download/. Only a minor fraction of the samples of the two datasets (3103 out of 58 235 about 0.05%) overlap; moreover, their annotated attributes are different. The model proposed in this work is able to learn to predict the union of the attributes of the two datasets, even if they are heterogeneous. To create the new dataset, we split each sample of the original datasets into as many samples as the number of its attributes. Thus, each sample of Cistrome was split into four samples, where each new sample has the same experiment metadata (data point) but a different attribute (label). Then, we merged all the samples thus obtained from the two original datasets. The attributes of the resulting dataset are extracted as indicated in Table 1. The Cell Line, Cell Type and Tissue information is retrieved from the corresponding attributes of Cistrome (when this is available) or from a combination of the Classification and Biosample term name from ENCODE, as an alternative. In the latter case: i) the attribute Classification specifies if the Biosample term name refers to a ‘cell line’ or a ‘tissue’. ii) Biosample term name’s content must be interpreted in the context of the classification scope. The combination of these two ENCODE attributes defines either a Cell Line or a Tissue; Cell Type is set to null. All the other attributes are mapped directly from ENCODE, with minimal renaming to better adhere to the terminology of the Genomic Conceptual Model (16).

**Table 1.** List of attributes considered from the Cistrome and ENCODE datasets and how they are mapped into GeMI’s output

Cistrome	ENCODE	GeMI
Cell Line		Cell Line
Cell Type	Classification	Cell Type
Tissue Type	Biosample term name	Tissue
	Assay Name	Technique
	Assay Type	Technique Type
	Target of Assay	Target
	Organism	Species
	Life stage	Life stage
	Age	Age
	Age units	Age units
	Sex	Sex
	Ethnicity	Ethnicity
	Health status	Disease
	Classification	Classification
	Investigated as	Feature

GEO database structure. GEO data model (https://www.ncbi.nlm.nih.gov/geo/info/overview.html) is composed of Samples, Series and Platforms records. In our work, we considered only Samples and Series. Any GEO series (GSE) represents an experiment that contains a collection of Samples (GSMs). We made the GEO repository searchable alternatively by GSE or GSM ids; specifically, we analyzed Samples’ metadata.

**Figure 2.**
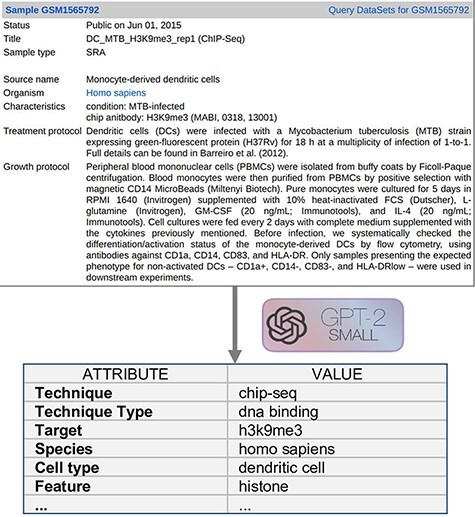
Example input document and possible output format.

Text Metadata Structure. Input descriptions are as shown on the upper area of Figure 2 and they correspond to the GEO metadata of the experimental Samples. For a bulk extraction of all the GEO samples’ metadata, we employed GEOparse Python library (https://github.com/guma44/GEOparse), searching by the GSM identifiers available in Cistrome and ENCODE experiments. As in Cannizzaro *et al.* (7), we transformed the semi-structured metadata table of each GEO Sample into a plain text string built as follows: keys (i.e. column definitions) are enclosed in squared parentheses and values (i.e. cell contents) are reported unchanged, in the form [name of the field]: metadata-free-text. The model can thus understand and exploit the patterns given by the structure of the table. The sample shown in Figure 2 results as follows:

[gsm]: gsm1565792 [title]: DC_MTB_H3K9me3_rep1 (ChIP-Seq) [sample type]: sra [source name]: Monocyte-derived dendritic cells [organism]: homo sapiens [characteristics]: condition: MTB-infected chip antibody: H3K9me3 (MABI, 0318, 13 001) [description]: …

For a limited number of ENCODE’s experiments, the corresponding GSM identifier was not available. For such cases, we filled the [characteristic]: and [description]: metadata fields of the input text by concatenating the content of ENCODE attributes Description, Biosample summary and replicates.library.biosample.description.

As part of the pre-processing, we changed the text into lowercase and we removed special characters such as ‘*’ and ‘_’. For complying with technological constraints of the GPT2 model, we removed all words that are >30 characters (the limit in this work corresponds to 400 tokens) and because—by empirical evaluation—most long words do not contain relevant information for the task.

### Model

The model has been fine-tuned according to two requirements:

- need to handle heterogeneous datasets by generating the union of all their attributes and
- need to predict each field independently from the others (for achieving better confidence of the predictions).

To this end, our resulting model differs from the fine-tuned GPT2 in Cannizzaro *et al.* (7), which needed two different versions of the models and generated all attributes at the same time, taking the previously generated attributes as input to generate the next attribute.

Here, instead, we propose a single fine-tuned model that can extract all the attributes provided from ENCODE and Cistrome; each attribute is extracted independently from the others. The output table generated one attribute at a time, exploiting the ‘task conditioning’ (10) meta-learning approach, which allows a model to perform as a multitask learner. In the case of GPT2, a multitask learning can be expressed as a conditional distribution P(output|input, task), where the task to be performed by the model is expressed in the form of text. In our context, we consider every attribute as a different task, so that a GPT2 model can extract a specific attribute when given the keyword ‘attribute_name’: as a task. An example formulation of fine-tuning training sample is given in Table 2.

**Table 2.** <BOS> <SEP> and <EOS> are special keywords that mean respectively ‘beginning of sentence’, ‘separated sentence’ and ‘end of the sentence’

Input	Task	Output
<BOS> [Input sentence] <SEP>	Cell line:	HeLa-S3 <EOS >

### Model interpretability

Deep learning models are able to find patterns and feature representation automatically. In our case, this allows to extract attributes that do not appear explicitly in the metadata of the experiment. For example, our model is able to infer the pair ‘Sex = Male’, just by knowing that the donor has prostate cancer. However, even when the model correctly extracts attributes, we cannot know if the result was achieved by exploiting wrong patterns. The AL framework offers support for such cases: the user must check prediction results and can potentially correct them. During this phase, it is critical that the user understands whether a given result is not only predicted correctly, but also whether it is predicted by exploiting a correct pattern/information. For such purpose, we resort to XAI techniques that allow the user to interpret the predictions of our model. Here, we focused on the generation of saliency maps, originally proposed for evaluating image classification (38), but recently applied to NLP (39). These are maps over the input that highlight the words that contributed the most to the extraction of given attributes. For predictions’ interpretation we have experimented three different approaches based on saliency maps, namely: LIME (Ribeiro *et al.* (39)), Attention (Bahdanau *et al.* (40)) and Gradient (Atanasova *et al.* (9)).

LIME (https://github.com/marcotcr/lime) showed to be impractical in real-time systems like ours. The Attention mechanism, typical of all transformer-based models (Vaswani *et al.* (13)), made it hard to find which combination of attentions was the best to interpret the model. Empirical evaluation of this approach led to disappointing results but present high efficiency in terms of compute time as during inference time of the model generates both the interpretations and the prediction.

**Figure 3.**
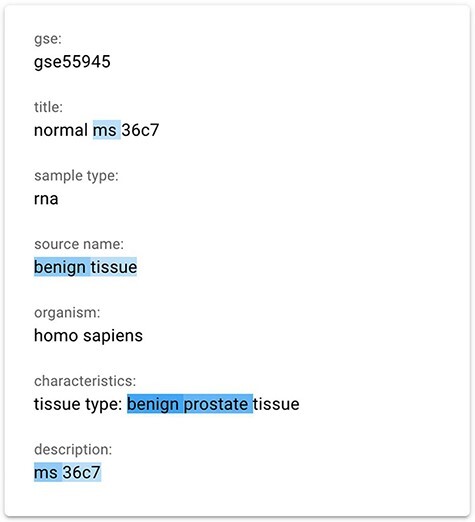
The gradient-based saliency map implemented in the GeMI tool. The words referring to the prediction of the ‘Sex’ attribute for the GSM1348947, ‘ms’, ‘benign tissue’, ‘benign prostate’ and ‘ms 36c7’ are highlighted because they are used by the model to predict the necessary fields.

We finally implemented a saliency map using the gradient-based technique InputXGrad (introduced by Kindermans *et al.* (15) and evaluated by Atanasova *et al.* (9)), exploiting the Ecco library (41) (https://github.com/jalammar/ecco) for our implementation with PyTorch (https://pytorch.org/). Differently from the Attention approach, generating saliency maps considering only the attentions layers of the model, the gradient-based technique considers the whole model. Empirically, we verified that the results of this method are similar or better than our implemented attention approach. Our assessment was also confirmed by the results provided in (9), where the InputXGrad approach is compared with LIME. This interpretation technique was selected for inclusion in the GeMI tool. One example application (related to the GSM1348947 sample) can be appreciated in Figure 3, where the ‘Sex = male’ was inferred from the information highlighted in blue, including the presence of benign prostate tissue.

### Active learning framework

Our AL framework adopts the model confidence as a metric to understand which data points are more informative for the model. For classification tasks, confidence is computed as the probability of the prediction. However, for text generation tasks, the definition is less trivial. In our scenario, a prediction is composed of a few words. Each token (i.e. a word or part of a word) of a text generated by a language model holds a probability that is conditioned by all the previous tokens. For computing the confidence of a single prediction, we thus combine a number of conditioned probabilities, with three possible approaches:

1. Multiply the probabilities of all the tokens. Note that GPT2 is a language model that uses sub-word tokens: for multi-token words, tokens after the first one exhibit a very high probability. In this way, when multiplying probabilities, second-to-last tokens do not lower the overall result.
2. Select the lowest probability among the tokens (characterizing the most critical part of the text).
3. Select the probability of the first token (considered the most informative part the text).

Surprisingly, Options 1 and 2 generate too many low values, even when the model predicts values correctly. On the contrary, the third (naive) option is more calibrated with the correctness of the prediction of the model and performs reasonably well; it was thus adopted in our AL framework.

Online Learning. Online learning is based on the idea of training a model as soon as new data become available. Implementing this mechanism in our framework has several advantages. First of all, it motivates the user by means of an instant visualization of the benefits of her work. Then, it diversifies the effort requested to the user, as explained next. In the considered scenario, new samples can be very similar to previously input data (especially when they belong to the same series). For this reason, a user could find that most errors of the predictor are repeated many times, leading to annoying interactions (labeling similar points does not give more information to the model). Online learning can mitigate this problem by prompting users to label similar samples only two times at most. As a drawback, after a while the model starts to forget the training data. To alleviate this drawback, every time a user labels a data point, this is added into a new dataset, which is used to augment our dataset when re-training the model.

**Figure 4.**
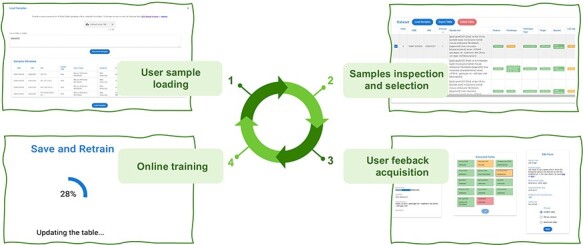
The four iterative phases of the AL framework.

Active Learning Framework Design. The GeMI AL framework is designed to be as much user-friendly as possible; our aim is to provide genomic researchers with a fun, interesting and useful tool, which keeps them motivated to a continued use. This will prospectively allow us to keep collecting the data that are needed to improve the performance of the model. The four iterative phases of the AL framework are shown in Figure 4:

1. The user provides a list of GSMs or GSEs identifiers. The model generates a table with the fields of the indicated GSM samples or of the GSM samples contained in the indicated GSE series.
2. The samples with predicted fields are sorted by ascending order of low confidence fields, i.e. the sample with the highest number of low confidence fields is shown first, as it the one that needs user editing the most.
3. The user edits the samples helped by the gradient saliency map (visualizing highlighted metadata text).
4. Once the editing session is over, the model is trained with the new data; the sample is removed from the table. At the end of the training, the table is generated again, showing a new first sample in the table.

The iteration is repeated: the user continues until the table ends or all the predictions show high confidence. We consider as highly confident each prediction with a confidence value above the green threshold value, which was set to 0.8. In the interface, we also use a yellow threshold value, set to 0.6, to signal lower confidence predictions. These threshold values were manually selected to provide for an intuitive interface. We leave to future work an investigation of i) the possible decoupling of the training threshold from the ones used in the interface and ii) the use of validation datasets and/or user feedback to tune these thresholds.

## Results

### Experiments

**Figure 5.**
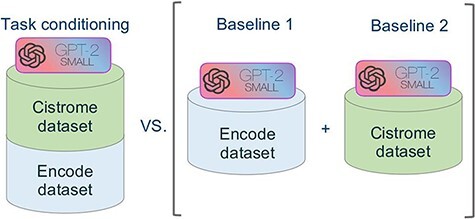
Schematic representation of the comparative experimental setting. On the right, separate models were trained on the ENCODE and Cistrome datasets (as presented in (7)). On the left, the task conditional setting presented in this work, employing the two training datasets together.

Figure 5 summarizes the approach of our experiment. In order to evaluate the performance of our novel model we used the model of Cannizzaro *et al.* (7) for building two baselines, trained respectively with the dataset of Cistrome and ENCODE. We evaluated our model separately against the two baselines, both in terms of accuracy and of inference time.

Setup. For the training phase, on the Google Colab platform https://colab.research.google.com, we used the Tensor Processing Units (TPU) v2 with 8 cores and 16-bit precision; for evaluation of the accuracy and the inference time we used the NVIDIA Tesla T4 with 16GB ram. The model was developed using the PyTorch https://pytorch.org library. The Transformer library from HuggingFace https://github.com/huggingface/transformers was exploited for the tokenizer and for downloading the pretrained GPT2. To setup the training with the TPU, we used the PyTorch Lightning library https://www.pytorchlightning.ai/. For training of our model and the baselines we used: i) for hyperparameters, CrossEntropy Loss Function; ii) an early-stop condition to avoid overfitting, with patient equal to 2 and min delta set to 0; iii) the Adam optimizer with a learning rate of 1e−4. In this work we used a batch size of 12. Note that, since we used a TPU with 8 cores in parallel and each core contains a replica of the model with a batch size of 12, the real batch size corresponds to 12 × 8 = 96. Finally, the used model is a fine-tuned GPT2 small architecture composed by 12 decoder layers, 12 head attentions and an embedding size of 768.

We initially set the learning rate according to previous work on data extraction (7) to be 0.001; then we reduced it by a factor of 10 (while also increasing the batch size from 5 right up to 96), based on manual inspection of the produced learning curves. We did not perform sweep over hyper-parameter settings, as our focus is not to optimize performance on a fixed training set, but rather to develop a system and interface capable of soliciting feedback from users to improve performance of the system as rapidly as possible.

**Figure 6.**
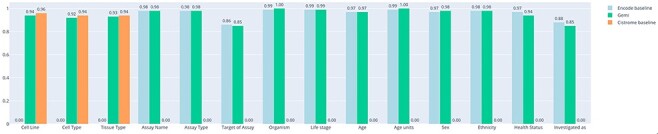
Bar plot representing the accuracy of experiments on the two separate baselines (trained on ENCODE or Cistrome data) and on our new model. On the *x*-axis we report all the attributes considered for prediction.

Evaluation. The results of the accuracy comparison are shown in Figure 6. Accuracy is computed considering the perfect match of the predicted strings; slight differences and synonyms are considered incorrect. Here we can appreciate that no relevant differences are measured in terms of accuracy. We can derive that the power of the GPT2 architectures depends on the quality of the dataset (which in this case is the same) and on the complexity of the architecture (in terms of number of layers, number of heads and dimension of the embeddings), as reported in literature. Moreover, we derive that the task conditioning mechanism does not affect the performance of the model in terms of accuracy. Table 3 reports the results of the comparison of the inference times required by the baseline models and the GeMI model. In the first experiment, we evaluated the Cistrome baseline against the GeMI model only considering the Cell Line, Cell Type and Tissue Type attributes. In the second experiment, we evaluated the ENCODE baseline against all other attributes. In total, we considered 1000 samples; GeMI required a significantly shorter time.

**Table 3.** Comparisons of inference time between (i) GeMI (with ENCODE-derived attributes) and the ENCODE baseline and (ii) GeMI (with Cistrome-derived attributes) and the Cistrome baseline

Model	Training time	Inference time per sample
GeMI (Cistrome attr. only)	10 h	0.27 s
Baseline Cistrome	2.49 h	0.38 s
GeMI (ENCODE attr. only)	10 h	0.49 s
Baseline ENCODE	0.58 h	0.81 s

### GeMI web application

**Figure 7.**
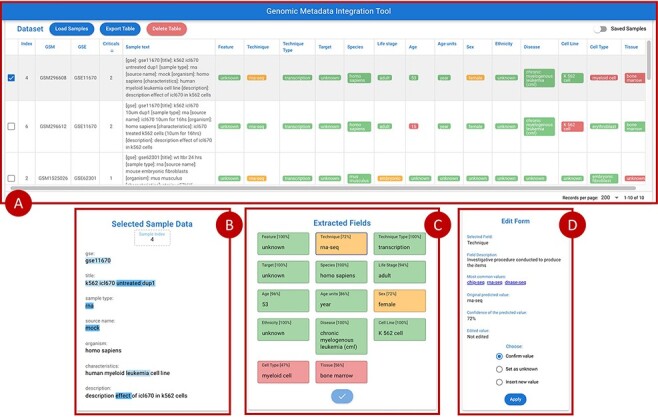
Overview of the GeMI interface, divided in four panels. Panel A represents loaded samples with original and predicted information. Panel B provides the gradient-based saliency map related to the sample selected in the table above. Panel C shows the predicted values for the selected sample, also reporting the for accuracy of the prediction. Panel D allows users to actively modify the prediction of the model and save the suggestions.

Figure 7 provides an overview of the main front-end elements provided in GeMI. Panel A shows the list of loaded samples; for each of them, we show the Sample (GSM) and Experiment (GSE) under analysis and the unstructured text that describes the sample on GEO. Then, several columns represent the attributes recognized by the tool, e.g. Feature, Technique and Sex. The system tries to fill all such attributes based on the input text. Predictions are marked as accurate when accuracy is > 0.8 (color code: green), to be verified when accuracy is between 0.8 and 0.6 (yellow), and probably wrong when accuracy is below 0.6 (red). From the top bar menu, users can access the samples loader, where samples are input as lists of GSMs or of enclosing GSEs either typed within a text box or uploaded by means of a text file. Additionally, they can export the results table in JSON or CSV format, delete the current uploaded samples and optionally save/resort to previously approved samples.

When a single row in Panel A is selected, Panel C (Extracted Fields) shows the detailed results obtained by the prediction for each attribute. When a specific field is selected here, Panel B shows the saliency map related to the model prediction of that specific attribute; blue highlight is used to inform users on which input text parts were used by the model to elaborate the prediction. Different shades, from lighter to darker, indicate how much (from weakly to strongly) the model exploited the highlighted information for the prediction. After inspection of this saliency map, users are invited to use the Edit Form in Panel D, where guidance (description and common values with external links) is provided to help make a decision. Here users can either (i) confirm the predicted value; (ii) set it as unknown (when the information should not be retrieved for the sample under scrutiny) or (iii) insert a new value manually. Once all the Extracted fields have been processed (i.e. they all turned green), the sample can be saved, thereby triggering a re-training session of the model and inserting the just processed sample within a list of annotated samples ready to be exported. Samples that have already been processed by users (i.e. for which the ‘Save Sample’ button has been pressed in the Panel C), can be recovered in the table of Panel A by using the switch button on the upper right corner of the panel.

We note that in GeMI we make the implicit assumption that the users are well-intentioned domain experts. Indeed, users who offer to spend their valuable time providing annotations are expected to be subject matter experts who see the value in doing so (to train an automated system to obviate the need for manual data extraction). Moreover, given sufficient training labels we expect the error rate for the system to approach that of the manual annotators.

### Basic use case

Suppose a user is interested in acquiring the metadata of GEO experiments studying the K562 cell line. Tools such as GEOmetadb (42) and the GEO Datasets Browser (https://www.ncbi.nlm.nih.gov/sites/GDSbrowser/) allow to select a list of GSMs (samples) or/and GSEs (series) that are related to the cell line of interest. This list can be loaded into GeMI; in this example, we suppose load the GSE11670 series. Once the GSE’s samples are loaded, the tool extracts the attributes for each of them and visualizes them in table of Panel A sorted by number of red attributes (i.e. with low confidence). Since the first considered sample (see GSM296608 as selected in Figure 7) requires user supervision, it is automatically loaded in Panels B, C and D of Figure 7. In Panel C, the user can select an extracted attribute (e.g. ‘Technique = rna-seq’ with 72% confidence), so that it is displayed in Panel B. Here, we show the words of the input text on which the model focused in order to predict the output attribute. Note, for instance, that the technique was inferred by using the information that i) the sample belongs to a particular experiment (GSE), as usually samples from the same experiment are also processed using a same assay; ii) the sample is of RNA type (thus, the assay is most likely an RNA sequencing); ii) the cell line was ‘untreated’ (from the title) or that the experiment was aimed at understanding the ‘effect’ of a specific medication (ICL670) on the cell line (from the description) and iii) the disease is leukemia (possibly because most of experiments on this kind of cancer in the training set were associated with this kind of assay).

By selecting the various attributes, we can observe that for some of them (e.g. species) the model has predicted a word exactly as it appears in the input text; in other cases, instead, the attribute has been inferred from other words contained in the text. Moreover, we observe that the attributes related to the characteristics of the donor (i.e. Life stage, Age, Age units, Sex and Ethnicity) are deduced from the cell line K562. When these attributes are selected, within the ‘Field Description’ of the Edit Form (Panel D), links to relevant external databases of cell lines are provided; these may be of help when assessing the accuracy of the extracted information. Specifically, we link back to the American Type Culture Collection (https://www.atcc.org/search) and to the Expasy Cellosaurus (43). This allows users to confirm that all the characteristics of the donor have been correctly deduced and that the model is confident with them. In this example, we observe that the model is not confident with the Tissue; a user check confirms that this is not the correct prediction. For correcting this field, users can use Panel D, ‘Insert new value’ option. For example, we insert ‘haematopoietic and lymphoid tissue’. By confirming our choices, the model is retrained. Once the new input is processed, the tool generates a new table of samples updated with this information. When there are no more errors in the table, users are invited to export the generated table with the dedicated button, for further inclusion in downstream pipelines or analyses.

This use case is also shown in the video provided to users within our evaluation form (described next). Please mind that the results reported in this description refer to the very first test of the use case on the system, before it went through several re-training cycles following different use sessions with the evaluators and other testers.

### Extended use case and tool comparison

In this section we describe an extended example of how GeMI can be used to annotate relevant datasets by providing standardized metadata information, which is then exploited for downstream biological analysis. For comparison, the same task is performed with an alternative state-of-the-art tool, i.e. OnASSiS (20, 44).

We selected a relevant dataset from GEO, corresponding to all Chip-seq data of *Homo sapiens*; in total, 6627 samples were extracted. For each sample, we pursue the computation of semantic annotations using the general format 〈information about cell type〉, 〈information about disease〉. Annotations performed with GeMI (proposed in this article) and the benchmark tool OnASSiS were compared. In GeMI, annotations were built by concatenating the ‘cell type’ and ‘health status’ attributes produced in output. In OnASSiS, the tool builds the desired annotations by selecting the ontological terms with minimum distance from the sample description; for this purpose, we used the Cell Ontology (45) for cell information and the Disease Ontology (46, 47) for disease information,

For the purpose of the comparison, we focused on samples dedicated to a small set of relevant factors, including both transcription factors (TFs) and histone modifications (HMs). Note that ‘factor’ field is automatically extracted by GeMI. For extracting comparable information from OnASSiS we instead resorted to Cistrome (11), by querying samples using GSM identifiers. Results were produced in the format 〈GSM, factor_list, semantic_annotation〉. Further numerical analysis was conducted to identify the sets of four TFs and HMs with the highest frequency in the dataset. Table 4 reports the results obtained for sets of four factors that include POL2RA (i.e. the gene that encodes for DNA-directed RNA polymerase II subunit RPB1) and CTCF (i.e. a transcriptional repressor involved in many cellular processes). The left part of the table shows the factor lists for which OnASSiS produced the richest sets of annotations (max 13), whereas the right part reports the top scores achieved by GeMI. As a relevant example, we focus on the list ‘POLR2A, CTCF, H3K4me1, H3K4me3’ (highlighted in bold in the table), which has 8 annotations in OnASSiS and 16 in GeMI, listed in Table 5. Note that annotations provided by OnASSiS can be multiple (without measures to indicate priority), e.g. the third annotation has both ‘colorectal cancer’ and ‘cancer’ values. They are less specific than the ones found by GeMI; for instance, in the second semantic annotation predicted by OnASSiS, the attribute ‘cell’ is very generic and does not provide meaningful information about the cell type. The coupling of this term with the ‘unknown’ attribute referring to the disease condition makes the semantic annotation unusable for any subsequent biological analysis.

**Table 4.** Attributes describing SARS-CoV-2 sequences in four data sources

**Factor list**	**#sem. annot**
Semantic annotations predicted by GeMI	
POLR2A, CTCF, H3K4me3, H3K27ac	16
**POLR2A, CTCF, H3K4me1, H3K4me3**	**16**
POLR2A, CTCF, H3K4me1, H3K27ac	15
POLR2A, CTCF, H3K4me3, H3K27me3	15
POLR2A, CTCF, H3K4me3, unknown	15
POLR2A, CTCF, H3K4me1, H3K36me3	14
POLR2A, CTCF, H3K4me3, H3K36me3	14
POLR2A, CTCF, H3K4me1, unknown	14
POLR2A, CTCF, H3K4me1, H3K27me3	14
POLR2A, CTCF, H3K27ac, H3K27me3	14
**Semantic annotations predicted by OnASSiS**	
POLR2A, CTCF, H3K27me3, H3K4me3	13
POLR2A, CTCF, H3K27ac, H3K4me3	11
POLR2A, CTCF, H3K27me3, H3K27ac	10
POLR2A, CTCF, H3K36me3, H3K4me3	8
POLR2A, CTCF, H3K27me3, H3K36me3	8
POLR2A, CTCF, MYC, H3K4me3	8
**POLR2A, CTCF, H3K4me1, H3K4me3**	**8**
POLR2A, CTCF, H3K4me1, H3K27me3	7
POLR2A, CTCF, H3K4me1, H3K27ac	7
POLR2A, CTCF, H3K27ac, H3K36me3	6

**Table 5.** List of semantic annotations for the set {POLR2A, CTCF, H3K4me1, H3K4me3}, using OnASSiS or GeMI

Source	Cell type	Disease
OnASSiS	Cell, erythroblast	Unknown
	Cell	Unknown
	Endodermal cell	Colorectal cancer, cancer
	Fibroblast	Unknown
	Lining cell, mesodermal cell	Cancer, chronic myeloid leukemia
	Lining cell	Unknown
	Lining cell	Neuroblastoma
	Progenitor cell	Unknown
GeMI	B lymphocyte	Unknown
	Embryonic stem cell	Healthy
	Embryonic stem cell	Unknown
	Epithelium	Breast cancer (adenocarcinoma)
	Epithelium	Cervical adenocarcinoma
	Epithelium	Hepatocellular carcinoma
	Epithelium	Mammary ductal carcinoma
	Epithelium	Prostate adenocarcinoma
	Epithelium	Unknown
	Erythroblast	Chronic myelogenous leukemia
	Erythroblast	Unknown
	Fibroblast	Unknown
	Keratinocyte	Unknown
	Lymphoblastoid	Unknown

Finally, the described annotations can be used as input in a more complex pipeline that aims, for example, to compare combinations of functional elements in multiple biological conditions (21). For conducting such analysis we can use a library for genomic data querying such as pyGMQL (48, 49) that allows to (i) extract the region data corresponding to the previously 6627 mentioned samples and (ii) verify which genomic regions are confirmed by at least one sample (i.e. a COVER(1,ANY) operation (50)), while grouping regions according to their factor and semantic annotation. Then, we identify functional states using ChromeHMM (51). Results for this analysis are shown in the Supplementary material (Figure S1), where the use of more accurate and richer semantic annotation such as the ones obtained through GeMI allows identifying a larger spectrum of functional states of chromatin, unless lost with OnASSiS annotations.

### User evaluation

Study Rationale. In line with other tools’ empirical evaluations (52), we asked 30 users (15 with a Biology background and 15 with a computer science background), to complete the survey available at https://forms.gle/VrAT5tiwHv7xZY299. After providing a brief introduction of GEO, GenoSurf (19) and the related metadata-integration problem, we described our proposed GeMI tool and provided a video tutorial (available at https://youtu.be/HLcDDIQ69YA) with more detailed explanations about the system. Subsequently, we asked users to solve six operational tasks using the GeMI interface and to evaluate their overall experience through four additional questions. As previously mentioned, GeMI is based on an AL framework; this introduces a bias in the evaluation because training data are continually updated and improved as a result of progressive user corrections. To mitigate such bias, we assigned different samples to each user who evaluated the system. Specifically, we manually selected a set of samples that covered different types of experiments and tissues/cell lines so as to minimize the semantic overlap between the task proposed to one evaluator and the task proposed to the previous evaluators.

**Figure 8.**
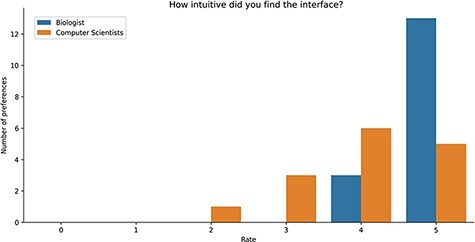
Bar plot of the answers to the question about the intuitiveness of GeMI according to the survey participant.

Study Results. Users were asked to evaluate the intuitiveness of the GeMI interface to solve various tasks. As reported in Figure 8, most users (18) rated this aspect with the maximum score 5/5; among these, 13 had a biological background and 5 a computer science background. Other 9 participants evaluated positively the intuitiveness of the tool (4/5 score), 6 being computer scientists and 3 biologists. Finally, three users evaluated the intuitiveness of the GeMI interface as low: two of them with a score of 2/5 and one with a score of 1/5 (all with a computer science background).

**Figure 9.**
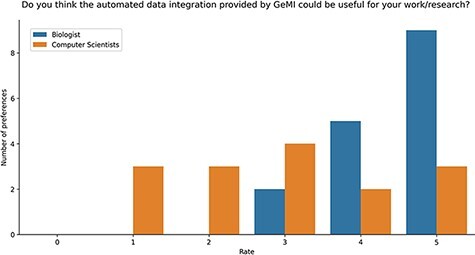
Bar plot of the answers to the question about the usefulness of GeMI for user’s future researches according to the survey participants.

Users were also asked if they deemed GeMI useful in their research. As shown in Figure 9, 12 users rated the tool with the maximum score 5/5; out of these, 9 had a biological background and 3 a computer science background. Then, 7 users rated GeMI’s usefulness with a 4/5 score; also in this case, the majority (5) was biologists, while the rest was computer scientists (2). Moreover, six people rated the tool’s usefulness with a score of 3/5 (four computer scientists and two biologists). Finally, six people rated GeMI’s usefulness as low (three with 2/5 and three with 1/5); all members of this group had a computer science background. No participant considered the interface useless. Results on the specific operational tasks confirmed that a considerable number of users found GeMI engaging, i.e. no user left the evaluation process before its end.

User Feedback. At the end of the survey, we asked participants to provide answers to an open-ended question: Do you have any suggestions for improving the GeMI tool? The collected indications were clustered into four main areas of improvement: (i) additions to the data model; (ii) changes to the user interface; (iii) management of user corrections and (iv) improvement of training and re-training phases. The most relevant ideas expressed in participants’ comments are summarized in Table 6; they have been fundamental to apply improvements to the framework and inspired several future work developments, discussed in the next{} section.

**Table 6.** Taxonomy of user-provided suggestions for improvement of GeMI

**Data model** Consider additional fields provided by GEO (e.g. platform) Allow a free schema, to include user-defined attributes (possibly, information about genotype or treatment) Add more possibilities to denote unknown attribute values: non specified, not applicable, none User interface Add information regarding the GSM in Panel B for user reference Reshape screen to provide a more comprehensive initial view of panels **User corrections** Aim to user corrections’ normalization by providing guidelines and value references (e.g. for life stage values) Provide feedback to the user when the sample to be annotated changes **Training and re-training phases** Return number and type of updated values after re-training Perform a stronger training of the model with gene expression-related datasets (now skewed toward ChIP-seq) Integrate the possibility of using parts of sentences suggested by users as ‘relevant for prediction’

## Toward GeMI version 2.0

Threats to the robustness of GeMI can arise from the degradation of its model; this can happen because of model overfitting or noisy input from user (malicious or sub-standard users). The first aspect has been discussed in the Setup section. As to the latter, we cannot measure how reliable a user who annotates the data is in general. However, in a future version of the system, we propose to (i) retain a validation dataset (which has not been used for training) and monitor the prediction performances on that dataset, verifying that the model does not collapse and (ii) use checkpoints, where the original model is retained while the new suggestions from users are only applied to a new instance of the model. In the latter case, different instances are compared to measure the trend of performances, checking that—based on the contribution of a specific user—the performances do not drop essentially. Optimization of this strategy will also be addressed.

We also plan to address several points from the feedback received from the study participants who left free-text comments. For example, a future version of GeMI will allow users to introduce new attributes to be extracted from the GEO description. It will also differentiate between different types of ‘unknown’ values, such as not present, not applicable, etc. We will also improve the guidance provided to users, supporting their annotation work with further tips, links to specialized ontologies (53) and reference tables and defining the set of valid values that a particular attribute can take. Finally, we will investigate whether it is possible to provide feedback to the domain expert on the extent to which their annotations improve the predictive performance of the model.

## Conclusions

We built a GeMI tool where the model can handle many heterogeneous datasets and predict each field independently. The inference process is faster than the baseline (7), while accuracy, recall and precision are comparable to the ones of the baseline. As to the proposed gradient-based interpretation mechanism, it is effective in interpreting the predictions of the model and allows a faster and easier identification of errors in the predictions. The employed AL framework requires few user annotations, while offering an intuitive and simple interface. In the future we aim to use domain-specific ontologies support, in order to simplify the management of synonyms and increase the same real-world concepts matching. In addition, we will evaluate alternative pretrained models, e.g., Bidirectional and Auto-Regressive Transformers (BART), which has been shown to achieve good results on summarization tasks (54).

Overall, the proposed approach and tool are a solid advancement in the user-aided metadata curation of genomic datasets. The simple output provided by GeMI backend suggests a straightforward embedding of our pipeline within more complicated data integration infrastructures, such as the one proposed by Bernasconi *et al.* (18) for extension of open data repositories (19). The prediction framework and the active learning-based interface are already being experimented within the context of new data types (e.g. extraction of mutation effects of SARS-CoV-2, the virus responsible for COVID-19) and we plan to generalize its use even further. With more extensive use of our tools, we will also conduct empirical studies aimed at quantifying the users’ gain when using them, e.g. by timing tasks performed by users.

## Supplementary Material

baac036_Supp

## Data Availability

The GeMI tool is openly available at http://gmql.eu/gemi/. The code is on GitHub at https://github.com/armando2603/GeMI.
