# Supplementary material for: GeMI: interactive interface for transformer-based Genomic Metadata Integration
Source: Database (Oxford). 2022 Jun 3;2022:baac036. doi: 10.1093/database/baac036 (PMC9216561; doi:10.1093/database/baac036)
Supplement: baac036_Supp [file baac036_supp.zip › SupplementaryMaterial.pdf]

Supplementary Material

Giuseppe Serna Garcia [1], Michele Leone [1], Anna Bernasconi [1], and Mark J. Carman [1]

[1]Dept. of Electronics, Information and Bioengineering, Politecnico di Milano

Comparison of GeMI vs Onassis

The matrix on the top left corner of Figure S1 shows the different functional states of chromatin, considering the histone modifications H3K4me1 and H3K4me3, the two transcription factors POLR2A and CTCF, as well as the CpG islands. The darker the blue tone in a cell of the matrix, the more intense the signal of the corresponding factor (column) in the related functional state (row).

In the figure we show two heatmaps; on the left the one obtained exploiting OnASSiS annotations, on the right the one with GeMI annotations. On the column we reported the semantic annotations (8 for OnASSiS, 16 for GeMI) indicated in Table 3 of the main manuscript. On the rows, we selected the portion of chromosome 17 related to the gene BRCA1 (43,044,295-43,170,245, according to the GRCh38 assembly).

As it can be easily spotted, the finer grain of GeMI annotations allow to identify more functional states that were unless lost with OnASSiS. This is particularly visible in the central area of the map (coordinates 43,124,600-43,126,600) and in the lower part of the map (coordinates 43,169,800-43,170,000), where several blue, purple, and pink states are now identified, capturing a signal of chromatin states 6 (enriched in H3K4me3), 8 (enriched in H3K4me1 and POLR2A), and 10 (CpG Islands). More details on the identification, semantic annotation and comparison of combinations of functional elements in multiple biological conditions can be found in [1].

References

[1] Leone M, Galeota E, Masseroli M, Pelizzola M. Identification, semantic annotation and comparison of combinations of functional elements in multiple biological conditions. Bioinformatics. 2021 12;38(5):1183-90.

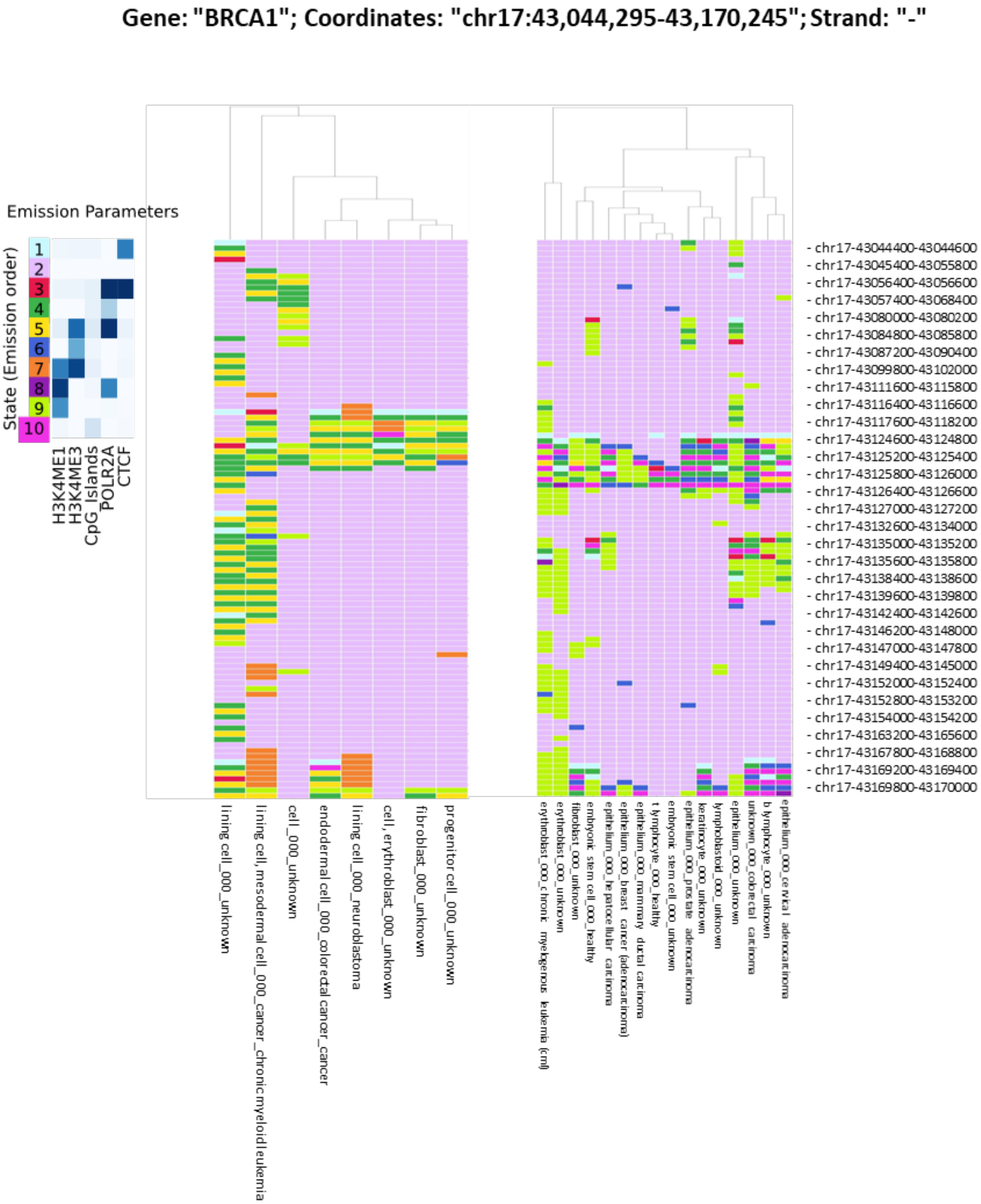

Figure 1: Chromatin functional states matrix and two heatmaps comparing chromatin states in the BRCA1 gene functionally annotated using Onassis (left) or GeMI (right).
